# Ventricular function assessment using an ultrafast spoiled gradient echo sequence with an intravascular blood pool contrast agent in pediatric patients

**DOI:** 10.1371/journal.pone.0318299

**Published:** 2025-01-31

**Authors:** Tayaba Miah, Rithvik Gunda, Gerald Greil, Mohammad Hussain, Qing Zou

**Affiliations:** 1 Division of Cardiology, Department of Pediatrics, UT Southwestern Medical Center, Dallas, Texas, United States of America; 2 Department of Radiology, UT Southwestern Medical Center, Dallas, Texas, United States of America; 3 Advanced Imaging Research Center, UT Southwestern Medical Center, Dallas, Texas, United States of America; University of Pisa, ITALY

## Abstract

**Background:**

Balanced steady-state free processing (bSSFP) MR sequence has long been considered the gold standard method for ventricular function assessment (VFA), and normal values are based on this acquisition. However, bSSFP sequence suffers from susceptibility artifacts due to scenarios such as cardiac implants. The T1-TFE sequence, also known as ultrafast spoiled gradient sequence, is less affected by such susceptibility artifacts. While it is unclear if T1-TFE sequence yields similar VFA results as the bSSFP sequence.

**Purpose:**

To validate if the T1-TFE sequence, with an intravascular blood pool contrast agent, yields similar results for VFA as the gold-standard non-contrast bSSFP approach, so that the T1-TFE sequence can be used for VFA when bSSFP approach fails.

**Methods:**

Two sets of images from two different sequences were utilized in this study. T1-TFE (with contrast) scans were used as one while bSSFP-derived images were used as the other. 37 pediatric patients were recruited into this study. Semi-automated software (cvi42) was used to segment and derive ventricular volumes. Image quality was objectively assessed by comparing signal-to-noise (SNR) and contrast-to-noise ratio (CNR) scores. Last, two expert readers provided a subjective analysis of image quality. Paired t-tests were used to assess significant differences in volumetric values (end-diastolic and end-systolic) between T1-TFE and bSSFP sequences. A Bland-Altman analysis evaluated potential bias and agreement between these sequences.

**Results:**

Ventricular function assessment via volumetric data analysis resulted in no statistically significant differences (*P >* 0.05), and high R^2^ values. SNR and CNR scores also presented with no statistically significant differences (*P >* 0.05), and nearly identical scores (SNR T1-TFE mean: 29.5 ± 3.1, SNR bSSFP mean: 28.8 ± 3.7, CNR T1-TFE mean: 28.8 ± 3.3, CNR bSSFP mean: 28.1 ± 4.0). Image quality assessment via expert subjective image analysis scores is consistent with the data. All Bland-Altman plots show good agreement and reveal no systematic bias or random error.

**Conclusion:**

T1-TFE sequences in combination with Ferumoxytol allow reliable ventricular function assessment and overcome the limitations of traditional bSSFP MR sequences in this context.

## Introduction

Cardiac MRI is considered the golden standard for ventricular function assessment (VFA) [1] as it is both highly reproducible and accurate in its definitions of endocardial and epicardial borders. VFA is critical in diagnosing and managing patients with different types of acquired and congenital heart diseases [2]. An essential part of VFA is ejection fraction (EF) measurement. EF has long been used as an indicator of cardiac strength and health and is a measurement of the percent of blood that leaves the ventricles with each contraction. In current clinical practice, cardiac cine MRI is usually obtained for VFA and is acquired by multi-slice 2D balanced Steady-State Free Precession (bSSFP) sequences [3]. The bSSFP sequence is known to provide excellent contrast between myocardium and blood pool with short acquisition time. Additionally, bSSFP sequence has both high signal-to-noise and contrast-to-noise ratios, also known as SNR and CNR, respectively. SNR is a measure of the image signal in a specific region in relation to the background. CNR is an objective measurement of the contrast between the tissue of interest and the surrounding tissue and/or background of the image. Higher SNR and CNR allow for increased image quality, leading to increased diagnostic accuracy. Furthermore, bSSFP sequences do not rely on the blood’s speed of flow, which can be especially advantageous when imaging patients with poor systolic function [4].

However, the bSSFP sequence suffers from banding artifacts (due to B0 inhomogeneity) and susceptibility artifacts (caused by metallic implants) [3]. These artifacts can lead to signal instability if they are near regions of heart, causing a significant decrease in image quality, and making it difficult to obtain accurate volume segmentation from these images [5]. In the case of implants such as pacemakers, cardiac cine MRI is then considered to be done using ultrafast spoiled gradient echo (Philips: T1-TFE; Siemens: TurboFLASH; GE: FGRE) sequence [6]. T1-TFE sequences vary from bSSFP sequences in the manner of the type of spoiling between the two. The bSSFP sequence is fully balanced, whereas T1-TFE sequences undergo gradient spoiling allowing to avoid the signal variations that arise from off-resonance effects, making it less susceptible to banding artifacts [7]. In contrast to bSSFP sequences, which have high flip angles that contribute to its sensitivity to off-resonance effects, spoiled gradient echo sequences, such as the T1-TFE sequence, has lower range flip angles and use generic T1-weighted contrast [8].

The traditional issue with the T1-TFE sequence for cardiac cine MRI is that the contrast between the myocardium and blood pool observed in the image is not comparable to bSSFP sequences. Furthermore, it is reported in [9] that the VFA obtained using T1-TFE is statistically significantly different from the results obtained using the bSSFP sequence. Additionally, due to lower contrast in T1-TFE imaging, specific values in previous studies, such as LV mass, have varied between T1-TFE and bSSFP imaging [10]. However, with the use of a contrast agent, T1-TFE sequences may have the potential to produce images of comparable quality and similar volumetrics to those of non-contrast bSSFP sequences. Ferumoxytol use is increasing in the setting of congenital heart disease [11, 12]. However, Ferumoxytol is known to cause significant T2 shortening resulting in significant degradation of the bSSFP sequence. As a result, some institutions are using T1-TFE sequences for VFA when using Ferumoxytol [13]. So, it is an important issue to delineate before the widespread adoption of T1-TFE sequence for VFA in the context of Ferumoxytol.

In this work, we validate that the T1-TFE sequence together with the intravascular blood pool contrast agent Ferumoxytol can provide similar VFA compared to the bSSFP sequence in pediatric patients, so that we can make sure that the T1-TFE sequence can be used for VFA when bSSFP approach fails such as in the scenario of cardiac implants.

## Methods

### Study population and sequences parameters

The study population consisted of 37 pediatric patients (aging from 5 months old to 21 years old, average age 10.4 years old and standard deviation 6.9; 26 males and 11 females; 19 single ventricle patients). These patients were prospectively enrolled into this study during the period of December 27, 2022 and June 27, 2023. Patients with a history of allergic reactions to Ferumoxytol or other intravenous iron preparations were excluded in this study. All data were collected on a 1.5T clinical MR scanner (Ingenia, Philips) using a 32-array dStream Torso coil (Philips HealthCare). This study was approved by the local institutional review board (STU032016-009). Written informed consent was obtained from all the participants or their legal guardians. The summary of the patient’s characteristics can be found in Table 1.

**Table 1:** Patient demographics and clinical indications for cardiac MRI.

Age (years, mean)	10.4
Male gender, N (%)	26 (70%)
Cardiac diagnoses, N (%)	
Hemitruncus	1 (2.7%)
Tetralogy of Fallot	9 (24.3%)
Hypoplastic Left Heart Syndrome	8 (21.6%)
Double Outlet Right Ventricle	4 (10.8%)
Double Outlet Left Ventricle	1 (2.7%)
Hypoplastic Right Heart Syndrome	1 (2.7%)
Bicuspid Aortic Valve	2 (5.4%)
Ventricular Septal Defect	1 (2.7%)
Tricuspid Atresia	2 (5.4%)
Pulmonary Valve Stenosis	1 (2.7%)
L-Transposition of the Great Arteries	2 (5.4%)
D-Transposition of the Great Arteries	1 (2.7%)
Coarctation of the Aorta	1 (2.7%)
AV Canal Defect	3 (8.1%)

Prior to injection of Ferumoxytol, the bSSFP sequence was used to obtain the 2D cardiac cine images with 30 cardiac phases, 2mm x 2mm x 2mm acquired voxel size and reconstructed to 1mm x 1mm spatial resolution. Sequence parameters of the bSSFP sequence include: TE/TR = 1.3ms/2.7ms, flip angle = 60, SENSE factor = 2. At least 15 minutes after Ferumoxytol contrast injection (dose of Ferumoxytol is 2mg/kg), the T1-TFE sequence was used to acquire the 2D cine images with 30 cardiac phases and 2mm x 2mm x 2mm acquired voxel size and reconstructed to 1mm x 1mm spatial resolution. Sequence parameters of the T1-TFE sequence include: TE/TR = 2.9ms/5.0ms, flip angle = 15, SENSE factor = 2. The FOV varies on each subject for both the two sequences and is dependent on the size of the subjects.

### Ventricular function assessment

The commercial software cvi42 was used to segment the ventricles and calculate the cardiac function, using left ventricle end-diastolic volume (LVEDV), left ventricle end-systolic volume (LVESV), left ventricle stroke volume (LVSV), left ventricle ejection fraction (LVEF), right ventricle end-diastolic volume (RVEDV), right ventricle end-systolic volume (RVESV), right ventricle stroke volume (RVSV), and right ventricle ejection fraction (RVEF). Short axis contours were obtained on the epicardial and endocardial border of the left ventricle, the right ventricle, and the myocardium at both the end-diastolic and end-systolic phases. The end-systolic (ES) and end-diastolic (ED) phases were determined to be where the smallest and largest ventricular volumes were. The ED/ES artificial intelligence phase detection program cvi42 was used to assist in these contours. Although contours done through ED/ES phase detection are generally completed with high accuracy, each contour was manually checked for inaccuracies, and if required, manual segmentation was completed.

### Image quality assessment

**Fig 1:**
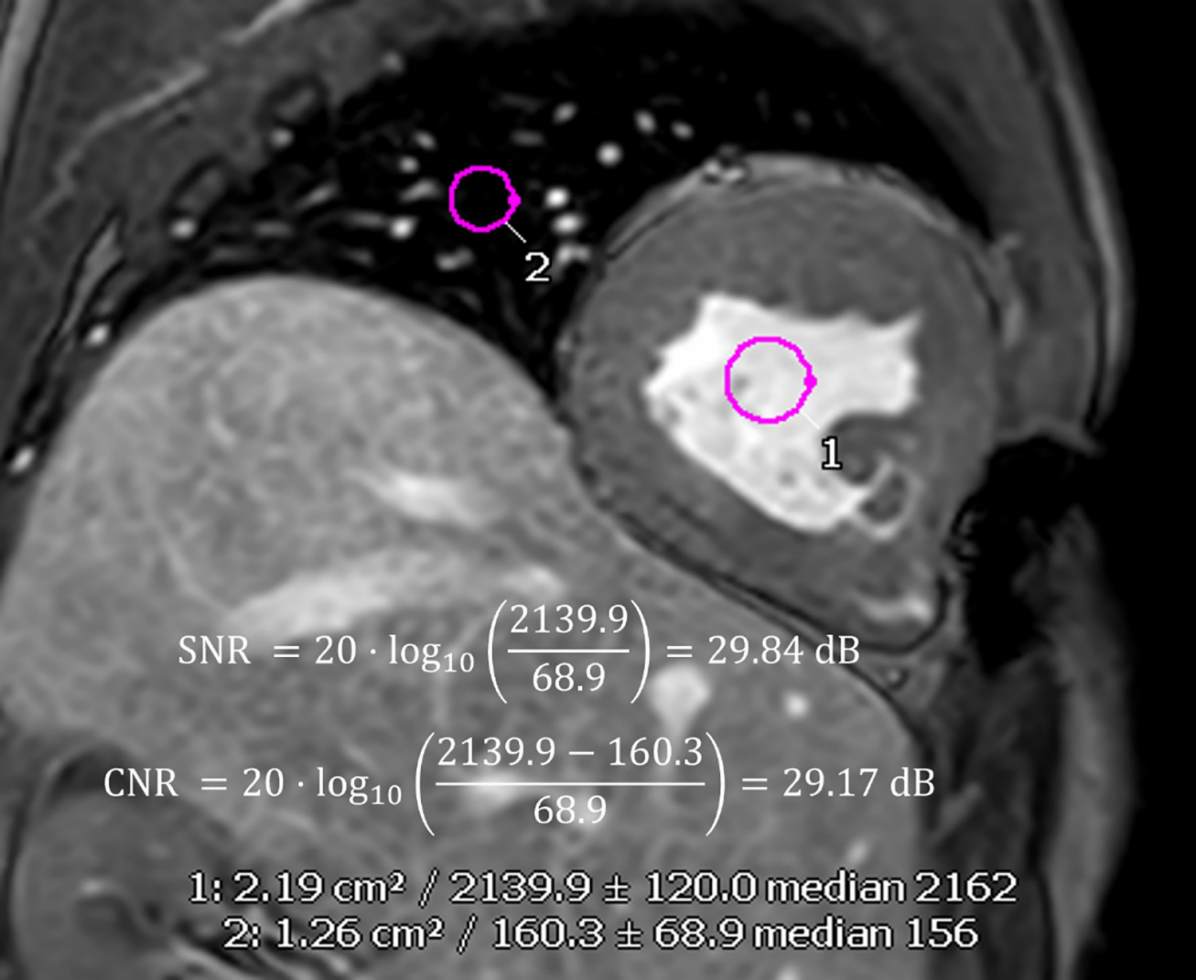
Illustration of blood-pool to lung SNR and CNR calculations in a mid-ventricle slice. The image is acquired using T1-TFE sequence. Two regions of interest (region 1 = blood pool, region 2 = lung) were manually chosen using circular region chosen tool in cvi42. Then the means, standard deviations as well as the medians of the selected regions will be reported, and these numbers are used for SNR and CNR calculations.

Both objective and subjective image quality assessments were performed in this work. For objective image quality assessment, we performed the apparent SNR and apparent CNR comparison between the images obtained from the two sequences. SNR and CNR values were calculated using cvi42. First, regions of interest were manually selected at three different slices of each patient’s scans (bSSFP and T1-TFE sequence scans): the apex, middle, and basal regions. Mid-ventricular slice was used, in addition to basal and apex slices, to decrease any potential partial volume effects, which may occur in basal and apical slices [14]. Following this portion of data collection, the SNR and CNR values in decibel for each patient’s scan were calculated using the average of the three values (one value from each slice) determined by the following definitions of SNR and CNR [15]:

$$SNR=20\cdot \log_{10}\left(\frac{\mu }{\sigma }\right)$$

where *μ* = mean of Region of Interest (ROI), and the blood pool region is chosen as ROI, *σ* = standard deviation of noise region.


$$CNR=20\cdot \log_{10}\left(\frac{\left|\mu_{A}-\mu_{B}\right|}{\sigma_{n}}\right)$$

where *μ_A_* = mean of ROI (blood pool area), *μ_B_* = mean of background region (lung region), *σ* = standard deviation of noise region. An illustration figure on SNR and CNR calculation for a mid-ventricle slice were shown in Fig 1.

**Fig 2:**
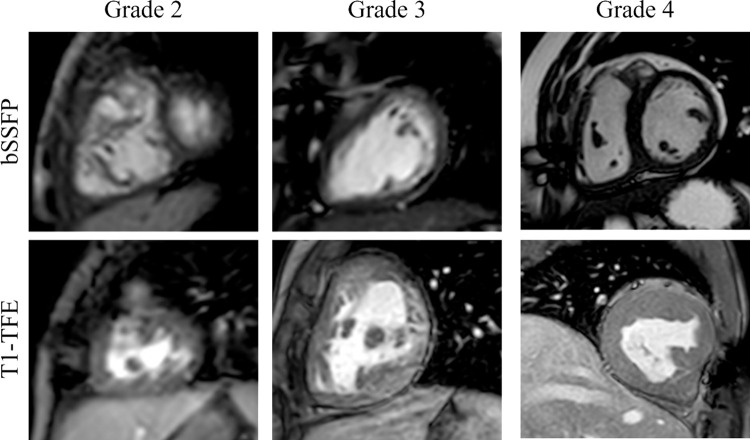
Examples of images with different image quality scores for both bSSFP sequence and T1-TFE sequence. In this study, all images receive scores no less than 2. So examples of grade 2, 3, and 4 are shown in this figure.

For subjective image quality assessment, image quality rating was done by two expert observers, who were blinded to the clinical details of the images, on a 5-point scale: grade 0: the ventricle(s) was (were) not visible; grade 1: the ventricle(s) was (were) visible with markedly blurred borders; grade 2: the ventricle(s) was (were) visible but with moderately blurred borders; grade 3: the ventricle(s) was (were) visible with mildly blurred borders; grade 4: the ventricle(s) was (were) visible with sharply defined borders. Under this scale, scores no less than 2 are considered clinically acceptable. Two cardiologists (with SCMR level 3 accreditation) who are trained in advanced cardiac imaging rated the image quality. Examples of images with different scores for the two sequences are shown in Fig 2.

### Statistical analysis

Statistical analyses were performed to compare the cardiac function obtained from the bSSFP sequence without contrast and the T1-TFE sequence with contrast. Paired t-tests [16] were performed to determine if there was a statistically significant difference between the volumetric (both end-diastolic and end-systolic) values obtained from both sequences. P values greater than 0.05 were considered not statistically significant. A Bland-Altman analysis [17] was conducted to evaluate any potential bias and determine the agreement between the T1-TFE and bSSFP sequences. R^2^ analysis [18] was also conducted in this study.

Paired t-tests and Bland-Altman plots were also used to analyze the SNR and CNR values as a part of the objective image quality assessment. Mean SNR and CNR scores were compared using a paired t-test.

## Results

### Ventricular function assessment

**Fig 3:**
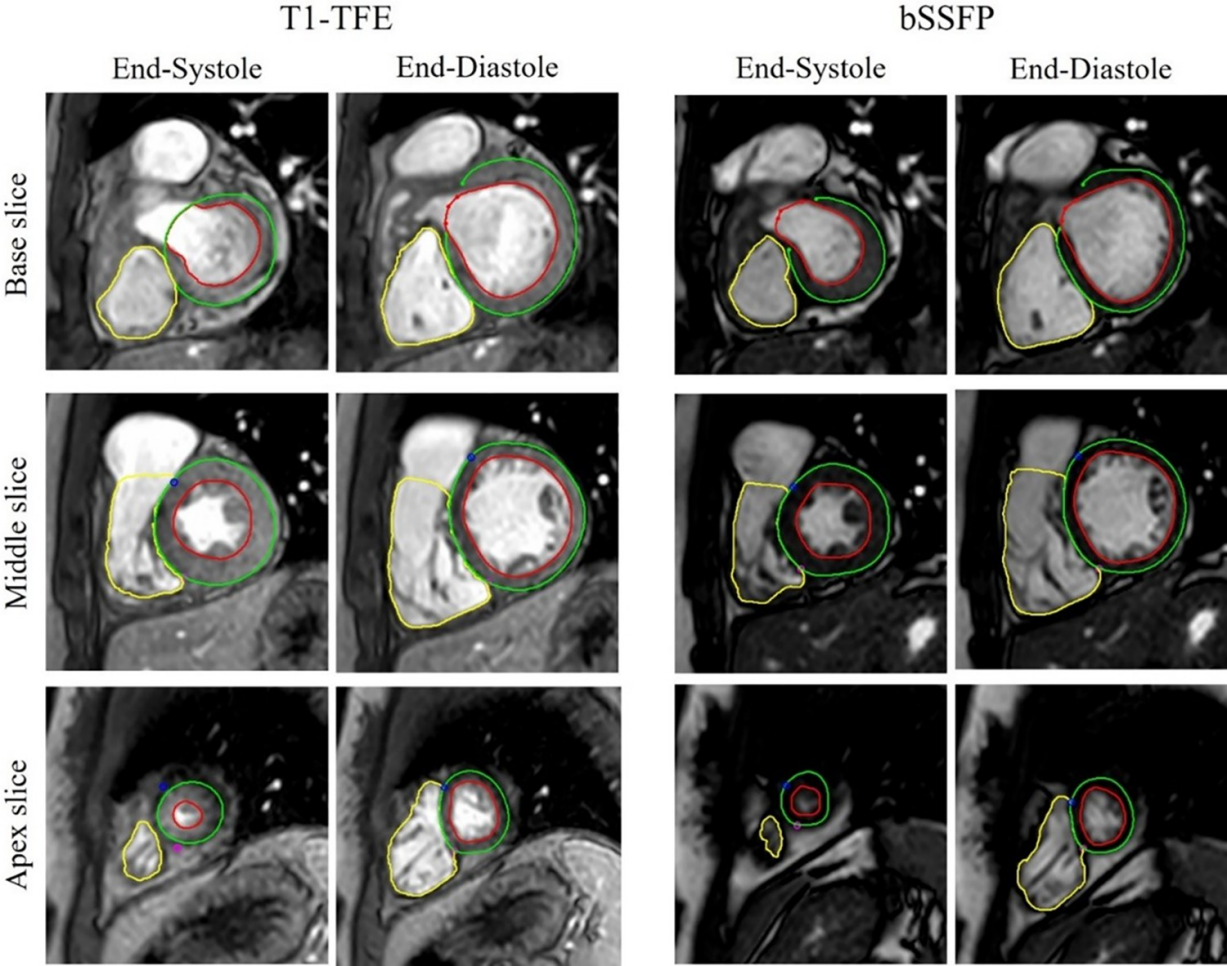
Visual comparison of the images and the segmentations on a 17-year-old female patient with tetralogy of Fallot status post repair. We showed the end-systolic and end-diastolic phases for the base slice, mid slice, and apex slice from both bSSFP sequence without contrast and T1-TFE sequence with Ferumoxytol.

First, the cine images from both sequences were compared visually. The images from both settings demonstrated reasonable image contrast and resolution, facilitating adequate segmentation. An example of the cardiac cine from the two sequences is shown in Fig 3. Throughout the segmentation process, there was no difficulty in delineating the endocardial, epicardial, or myocardial borders of images acquired from either sequence.

**Fig 4:**
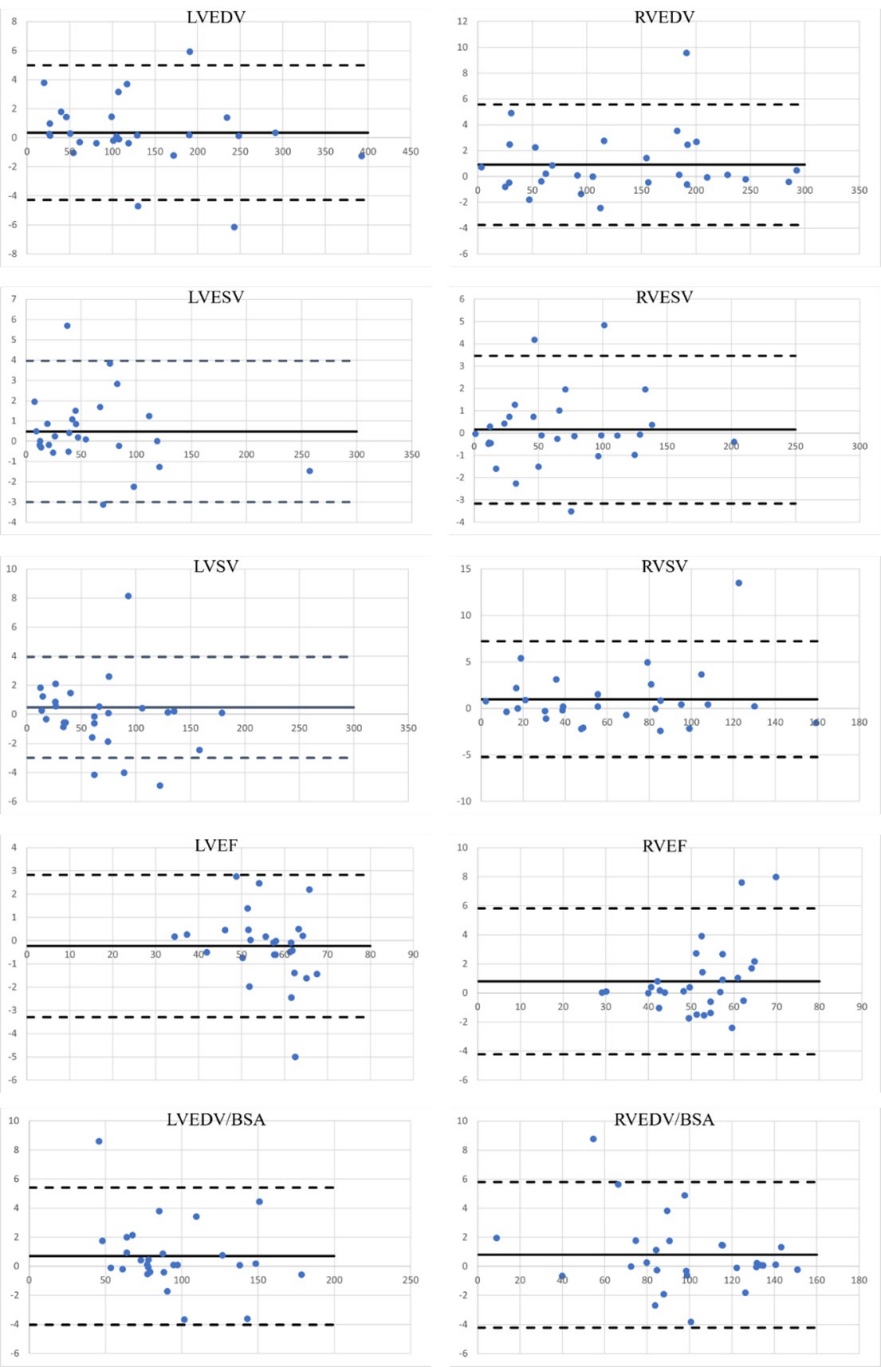
Statistical analysis for LVEDV, LVESV, LVSV, LVEF, LVEDV/BSA, RVEDV, RVESV, RVSV, RVEF, and RVEDV/BSA obtained from the bSSFP cine images and the T1-TFE cine images. Bold lines indicate the bias, and dashed lines indicate the limits of agreement within a 95% confidence interval. Here, we plotted the Bland-Altman plots for these values. The statistical results showed that there is agreement and no significant difference between the cardiac function obtained using bSSFP and T1-TFE with contrast agent.

The comparison of the LV and RV function from the two sequences is shown in Table 2, with the paired t-test results also shown in Table 2. The R^2^ values and the Bland-Altman plots shown in Fig 4 provided supportive evidence for the conclusion that the T1-TFE sequence together with the intravascular blood pool contrast agent Ferumoxytol can provide similar VFA compared to the bSSFP sequence. All LV and RV values showed a strong correlation between measures with the lowest R^2^ value of 0.952 (RVEF). The Bland-Altman plots demonstrate random scatter patterns, with the absence of specific patterns, indicating no systematic difference across measurement magnitudes. A comprehensive review of all included Bland-Altman plots shows excellent agreement between the bSSFP sequence without contrast and the T1-TFE sequence with Ferumoxytol with narrow limits of agreements across all volumetric measures.

**Table 2:** Mean ± SD of LV and RV dimensions based on acquisition with bSSFP and T1-TFE.

	LV				RV			
	bSSFP	T1-TFE	p-value	R^2^ value	bSSFP	T1-TFE	p-value	R^2^ value
EDV (ml)	126.6 ± 90.7	126.3 ± 91.2	0.449	0.999	130.6 ± 82.7	129.7 ± 82.6	0.053	0.999
ESV (ml)	58.9 ± 51.3	58.4 ± 51.7	0.176	0.999	66.8 ± 48.8	66.6 ± 48.6	0.637	0.999
SV (ml)	67.7 ± 45.1	67.8 ± 45.5	0.901	0.997	63.8 ± 40.0	62.8 ± 39.6	0.111	0.994
EF (%)	55.6 ± 8.3	55.8 ± 8.7	0.437	0.970	51.9 ± 10.4	51.1 ± 9.4	0.081	0.952
EDV/BSA (ml/m^2^)	93.3 ± 33.4	92.6 ± 34.0	0.148	0.995	98.9 ± 32.4	98.1 ± 33.2	0.113	0.995

Note. LV = Left ventricle, RV = Right ventricle, EDV = End-diastolic volume, ESV = End-systolic volume, SV = Stroke volume, EF = Ejection fraction, BSA = Body surface area.

### SNR and CNR comparison

As detailed in Table 3, we calculated the blood-pool to lung and blood-pool to myocardium SNR, as well as blood-pool to lung, blood-pool to myocardium and myocardium to lung CNR for images acquired using both the bSSFP sequence without contrast agent and T-TFE sequence with contrast agent. The paired t-test reveals no significant difference in the SNR values between the T1-TFE and bSSFP sequences, when contrast agent is applied for the T1-TFE sequence. This finding indicates that both imaging techniques perform comparably in terms of SNR. For the CNR values, there is no significant difference for blood-pool to lung CNR for the two sequences, while the blood-pool to myocardium CNR for the bSSFP sequence is better than the T1-TFE sequence, and the myocardium to lung CNR for the T1-TFE sequence is better than the bSSFP sequence. The better blood-pool to myocardium CNR for the bSSFP sequence indicates better endocardial borders for the images acquired from the bSSFP sequence. But from clinical standing point, the readers can still segment the blood-pool from the images acquired using the T1-TFE sequence with contrast agent. The better myocardium to lung CNR for the T1-TFE sequence indicates better epicardial borders for the images acquired from the T1-TFE sequence with contrast agent.

**Table 3:** Statistical analysis of signal-to-noise and contrast-to-noise ratio.

	SNR		CNR		
	Blood-pool to lung	Blood-pool to myocardium	Blood-pool to lung	Blood-pool to myocardium	myocardium to lung
T1-TFE	29.5±3.1	34.6±4.6	28.8±3.3	28.8±3.2	21.8±2.6
bSSFP	28.8±3.7	35.3±4.1	28.1±4.0	32.2±3.8	15.6±5.3
p-value	0.212	0.321	0.277	<0.05	<0.05

### Subjective image assessment

**Fig 5:**
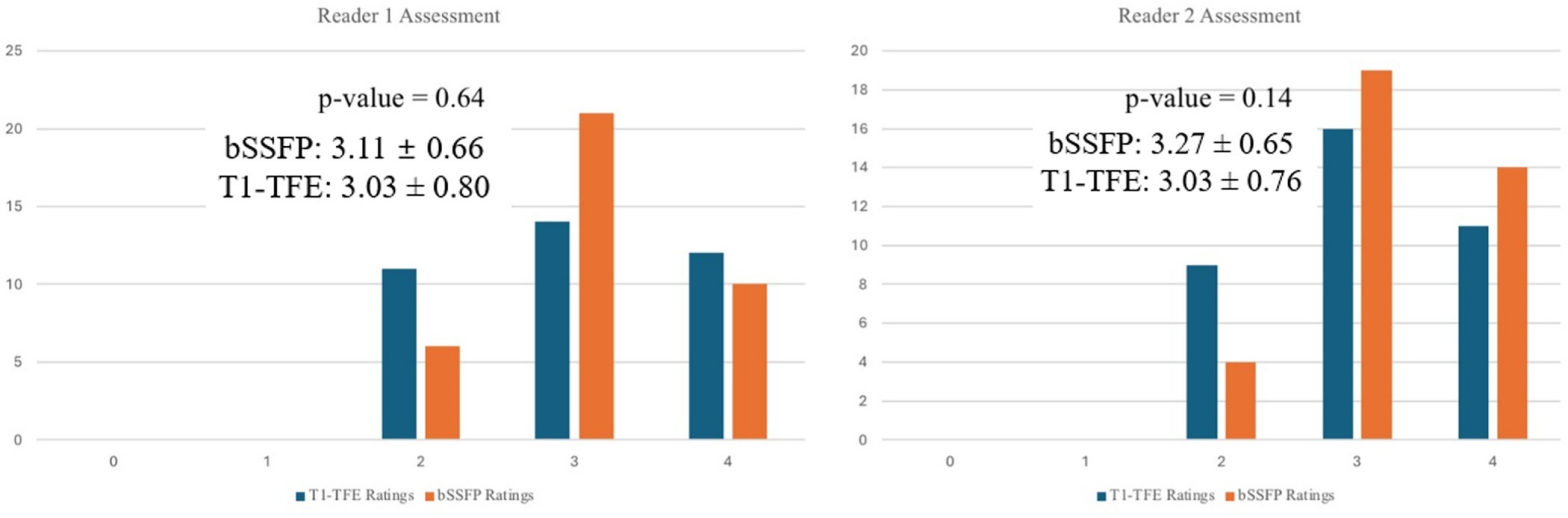
This figure presents a graphical representation of the subjective image assessment done by two readers. Their respective p-values from paired t-test are included. The left side provides Reader 1’s ratings, and the right side provides Reader 2’s ratings.

The results of the subjective image analysis, as illustrated in Fig 5, indicate that the overall image quality for both T1-TFE sequence with contrast agent and bSSFP sequence ranged from moderate to excellent. This assessment was consistent across both readers, demonstrating that both imaging sequences are capable of producing high-quality images suitable for clinical evaluation. Specifically, the images obtained from the bSSFP sequences received an average rating of nearly 3, with Reader 1 assigning a mean score of 3.11 ± 0.66 and Reader 2 assigning a mean score of 3.27 ± 0.65. Similarly, the T1-TFE sequence images with Ferumoxytol were rated with average scores of 3.03 ± 0.80 and 3.03 ± 0.76 by Readers 1 and 2, respectively. Paired t-test was performed to compare the image quality scores from each reader. The p-value for the image quality scores of the two sequences from Reader 1 is 0.64; and the p-value for the image quality scores of the two sequences from Reader 2 is 0.14. These results suggest that both T1-TFE with Ferumoxytol and bSSFP sequences offer comparable image quality, with neither sequence showing a significant advantage over the other in subjective evaluations.

## Discussion

In this study, we proposed that VFA can be accurately conducted using an ultrafast spoiled gradient echo sequence (T1-TFE) with the intravascular blood pool contrast agent, Ferumoxytol, in a group of pediatric patients. To assess the feasibility of the proposed cine imaging protocol, CMR data was gathered from 37 pediatric patients. MRI data was acquired using the breath-hold technique to generate the clearest images possible. The study utilized two scans: one using the bSSFP without contrast and the other using T1-TFE with Ferumoxytol contrast. Three analysis methods were employed to evaluate the effectiveness of the T1-TFE sequence with Ferumoxytol contrast for VFA. Ejection fraction (EF%), end-diastolic volume (EDV), end-systolic volume (ESV), and stroke volume (SV) were calculated. These values were collected for both the left and right ventricles for each patient using cvi42, which is a semi-automated software. Two critical factors in assessing MR image quality are SNR and CNR. SNR and CNR were calculated by selecting two regions of interest, one from the left ventricular area and one from the right ventricular area, and a "noise" region (the lung region) on each image’s basal, apical, and mid-ventricular slices. We observed no significant differences and high levels of agreement between the two sets of volumetric data derived from the bSSFP and T1-TFE sequences. The SNR values also showed no significant difference between the two sets of images. This comparability implies that the choice between T1-TFE and bSSFP can be made based on specific clinical requirements, patient condition, or the particular imaging scenario, without concern for compromising SNR. Besides, the blood-pool to lung CNR for the two sets of images are also comparable. While bSSFP shows better endocardial borders and T1-TFE shows better epicardial borders. The CMR readers finds both two sets of images can be used to segment the blood-pool and myocardium without any issue. This result reinforces the versatility of both sequences in various clinical settings, particularly in applications when patients with devices. Last, the subjective analysis provided evidence that T1-TFE images can provide acceptable image quality and that they are comparable to bSSFP-derived images when determining VFA. The consistency of the ratings between the two readers further reinforces the reliability of these findings, indicating that the perceived image quality is stable and reproducible across different observers. This level of consistency is crucial for clinical applications, where the ability to produce reliable and high-quality images can significantly impact diagnostic accuracy and patient outcomes.

Previous studies have shown significant differences in ventricular volumetric data between the bSSFP sequence and the ultrafast spoiled gradient echo sequence [9, 10]. However, these studies were performed without the use of an intravascular blood pool contrast agent. This study demonstrates that when used in combination with Ferumoxytol as a contrast agent, T1-TFE imaging can produce VFA results similar to bSSFP sequencing.

Please note that our study is targeted to patients with congenital heart disease (CHD). The rising use of Ferumoxytol for this indication makes this study important in confirming the applicability of current reference values that use b-SSFP sequences.

## Conclusion

This study investigated if ventricular function assessment could be effectively done using an ultrafast spoiled gradient echo sequence with an intravascular blood pool contrast agent. The T1-TFE sequence with Ferumoxytol as a contrast agent was assessed for this purpose and was compared to the traditional non-contrast bSSFP sequence that is typically used for VFA. We found that T1-TFE with ferumoxytol does produce comparable results to bSSFP in its ability to assess ventricular function and no significant difference between the two throughout the measures conducted in the group of pediatric patients. We propose that the T1-TFE approach in this context can continue to use existing reference values in this population.
